# Space Constrained Homology Modelling: The Paradigm of the RNA-Dependent RNA Polymerase of Dengue (Type II) Virus

**DOI:** 10.1155/2013/108910

**Published:** 2013-08-06

**Authors:** Dimitrios Vlachakis, Dimitrios Georgios Kontopoulos, Sophia Kossida

**Affiliations:** Bioinformatics & Medical Informatics Team, Biomedical Research Foundation, Academy of Athens, Soranou Efessiou 4, 11527 Athens, Greece

## Abstract

Protein structure is more conserved than sequence in nature. In this direction we developed a novel methodology that significantly improves conventional homology modelling when sequence identity is low, by taking into consideration 3D structural features of the template, such as size and shape. Herein, our new homology modelling approach was applied to the homology modelling of the RNA-dependent RNA polymerase (RdRp) of dengue (type II) virus. The RdRp of dengue was chosen due to the low sequence similarity shared between the dengue virus polymerase and the available templates, while purposely avoiding to use the actual X-ray structure that is available for the dengue RdRp. The novel approach takes advantage of 3D space corresponding to protein shape and size by creating a 3D scaffold of the template structure. The dengue polymerase model built by the novel approach exhibited all features of RNA-dependent RNA polymerases and was almost identical to the X-ray structure of the dengue RdRp, as opposed to the model built by conventional homology modelling. Therefore, we propose that the space-aided homology modelling approach can be of a more general use to homology modelling of enzymes sharing low sequence similarity with the template structures.

## 1. Introduction

3D structural information provides invaluable insights into the organization, mode of action, folding, and utterly function of a given protein. The 3D structure of proteins is usually experimentally determined using X-ray crystallography, NMR, or microscopy [1]. However, protein expression, purification, and crystallization are quite tedious experiments with uncertain outcomes and success rates. Thus, the next best thing to experimentally determine the structure of a protein is done via state of the art computational techniques and mainly homology modelling [2].

Homology modelling is the current leading technique for *in silico *predicting the three-dimensional structures of proteins. However, the quality of the predicted structures is only limited to the homology shared between the query protein and the chosen template structure [3]. 

Conventional homology modelling methods are comprised of the following steps [4–6]: first an initial partial geometry specification, where an initial partial geometry for each target sequence is copied from regions of one or more template chains, secondly, the insertions and deletions task, where residues that still have no assigned backbone coordinates are modelled. Those residues may be in loops (insertions in the model with respect to the template), they may be outgaps (residues in a model sequence which are aligned before the C-terminus or after the N-terminus of its template) or may be deletions (regions, where the template has an insertion with respect to the model). For this study outgaps have not been included in the homology modelling process. Third step is the loop selection and side chain packing, where a collection of independent models is created. Last step is the final model selection and refinement one, where the final models are scored and ranked, after they have been stereochemically checked for persisting errors.

In the present work, a use case was sought that would be impossible to model using the previously described conventional homology techniques in an effort to put to the test and apply our novel proposed homology modelling technique. In this direction an example from the highly mutagenic field of RNA viruses was selected. In particular, we chose to model the three-dimensional structure of the RNA-dependent RNA polymerase (RdRp) of Dengue (type II) virus by using the crystal structures of other polymerases of the *Flaviviridae* family as templates and by applying our novel homology modelling approach. The novel approach takes advantage of the 3D space corresponding to ligands, substrates, and the size and shape of the template structures (by creating a mould made of dummy atoms) in an effort to restrain the folding of the target protein. In this way our proposed modelling technique manages to overcome fundamental limitations in the homology modelling methodologies that originate from the low sequence identity shared between the query (Dengue polymerase) and the RdRps used as templates.

The viral family *Flaviviridae* comprises the genera *Flavivirus*, *Pestivirus*, and *Hepacivirus *and includes numerous important human and animal pathogens [10]. The small, enveloped virions of the different members of the *Flaviviridae* family contain a single-stranded, positive-sense RNA genome of about 9.5–12.5 kb. The genome consists of a single, long open reading frame (ORF), which is flanked by untranslated regions (UTRs) at the 5′ and 3′ ends. Recent studies on subgenomic pestivirus and flavivirus RNA replicons have revealed that the nonstructural (NS) proteins, which are encoded by the C-terminal part of the polyprotein, play a crucial role in viral RNA replication [11]. Accordingly, these proteins are assumed to form replication complexes in conjunction with genomic RNA and possibly with other cellular factors.

The NS5 proteins of hepatitis C virus (HCV), bovine viral diarrhoea virus (BVDV), and Dengue flavivirus type II have been attributed a RNA-dependent RNA polymerase (RdRp) function and thus constitute very good targets for the drug design approach [12]. Sequence alignments of viral RNA-dependent polymerases (reverse transcriptases and RdRps) have identified several conserved sequence motifs that are important for biological functions [8]. So far, the crystal structures of RdRps from various RNA viruses have been determined, including the RdRp from reovirus [13], calicivirus [14], poliovirus [15], Φ6 [16], hepatitis virus (HCV) [7], and bovine viral diarrhea virus (BVDV) [8]. All structures follow the generic shape of a right hand with “fingers,” “palm,” and “thumb” domains. Those structures shed light on key aspects of the biology of RdRps and confirmed the hypothesis that RdRps share a common architecture and mechanism for polymerase catalysis [17]. In particular, comparison of the crystal structures of the RdRps of HCV and BVDV, which belong to the *Flaviviridae* family, revealed that the “fingers” and “palm” domains are structurally similar forming a conserved “core” common to other polymerases, whereas the “thumb” domain is more variable [18]. Dengue is the most important mosquito-borne viral disease affecting humans with a distribution comparable to that of malaria. Approximately 2.5 billion people are living in areas at risk for epidemic transmission [19]. The usual outcome of this type of disease is the Dengue haemorrhagic fever (DHF) and Dengue shock syndrome (DSS) resulting in blood circulation interruption. 

Overall, it was found that the model derived from conventional homology modelling was poor in quality and structurally incapable for the *in silico *processing of the ssRNA fragment since it bared numerous clashes between the oligonucleotide and the protein's backbone atoms. On the contrary, the model derived from our enhanced homology modelling methodology that utilises a series of spatial constrains successfully incorporated all RdRp conserved motifs and was almost identical to the existing Dengue (type II) X-ray crystal structure [20]. Therefore, we propose that the space-aided 3D modelling approach is beneficial to the homology modelling of enzymes sharing low sequence similarity with the template structures. Finally, the stand-alone application “space-mould” was developed in an effort to facilitate and automate the incorporation of the 3D spatial constrains that are required by our proposed modelling technique. The software is available as a GNU licensed scientific freeware package at http://www.bioacademy.gr/bioinformatics/space/index.html. 

## 2. Methods

All computations and simulations were carried out on an Intel P4-based Microsoft Windows XP workstation mainly using MOE 2005.03 Package [21] unless otherwise stated. 

### 2.1. Sequence Analysis

The amino acid sequence of dengue polymerase was obtained from the GenBank database (accession no. NC_001474, entry name: dengue virus type 2, complete genome) [22]. Secondary structure predictions were performed using the NPS (Network Protein Sequence Analysis) web server [23]. The Gapped-BLAST [24] through NCBI was used to identify homologous structures by searching the protein structure database RCSB [25, 26]. The search detected the crystal structures of HCV [27] and BVDV polymerases [8]. These structures were subsequently used as templates for the homology modelling of the dengue polymerase. 

### 2.2. 3D Modelling

The 3D modelling of the Dengue RdRp was performed using the MOE package through the built in homology modelling module. The RCSB entries 1NB7 and 1S48 corresponding to the crystal structures of the HCV [7] and BVDV [8] RdRps, respectively, were used as templates for this purpose. Full coordinates from the template structures were transferred to the target protein for regions with sequence identity, whereas backbone coordinates were utilised for regions with sequence similarity. For domains corresponding to deletions or insertions in the sequence alignment a Boltzmann-weighted randomized modelling procedure [28] was employed. This procedure was combined with geometric scoring criteria for the proper handling of insertions and deletions as reported in Fechteler et al. [29]. The produced models were evaluated by a residue packing quality function, which depends on the number of buried nonpolar side chain groups and on hydrogen bonding [21].

Due to low sequence identity, an enhanced approach to conventional homology modelling process was applied. The novel approach involves the exploitation of common 3D space on the template structures. For this purpose, the conformational space corresponding to the ssRNA, UTP, and rNTP tunnel regions was first calculated and subsequently filled by alpha spheres [21]. The set of alpha spheres cloud was then used as a set of user-defined restraint to the homology modelling process.

### 2.3. Model Refinement

The initial models were further optimized by energy minimization using the conjugate gradient method as implemented within MOE and the CHARMM22 forcefield [30]. The energy minimization was performed until the gradient was less than 10^−5 ^kJ/(moL Å) with a distance-dependent dielectric constant of 4 to approximate solvent effects.

The quality of the final models was assessed using the PROCHECK suite of programs [31].

### 2.4. Molecular Electrostatic Potential (MEP)

Electrostatic potential surfaces were calculated by solving nonlinear Poisson-Boltzmann equation using finite difference method [21] as implemented in the Pymol Software [32]. The potential was calculated on grid points per side (65, 65, 65) and the “grid fill by solute” parameter was set to 80%. The dielectric constants of the solvent and the solute were set to 80.0 and 2.0, respectively. An ionic exclusion radius of 2.0 Å, a solvent radius of 1.4 Å, and a solvent ionic strength of 0.145 M were applied. AMBER99 [33] charges and atomic radii were used for this calculation.

## 3. Results and Discussion

### 3.1. Sequence Alignment

**Figure 1:**
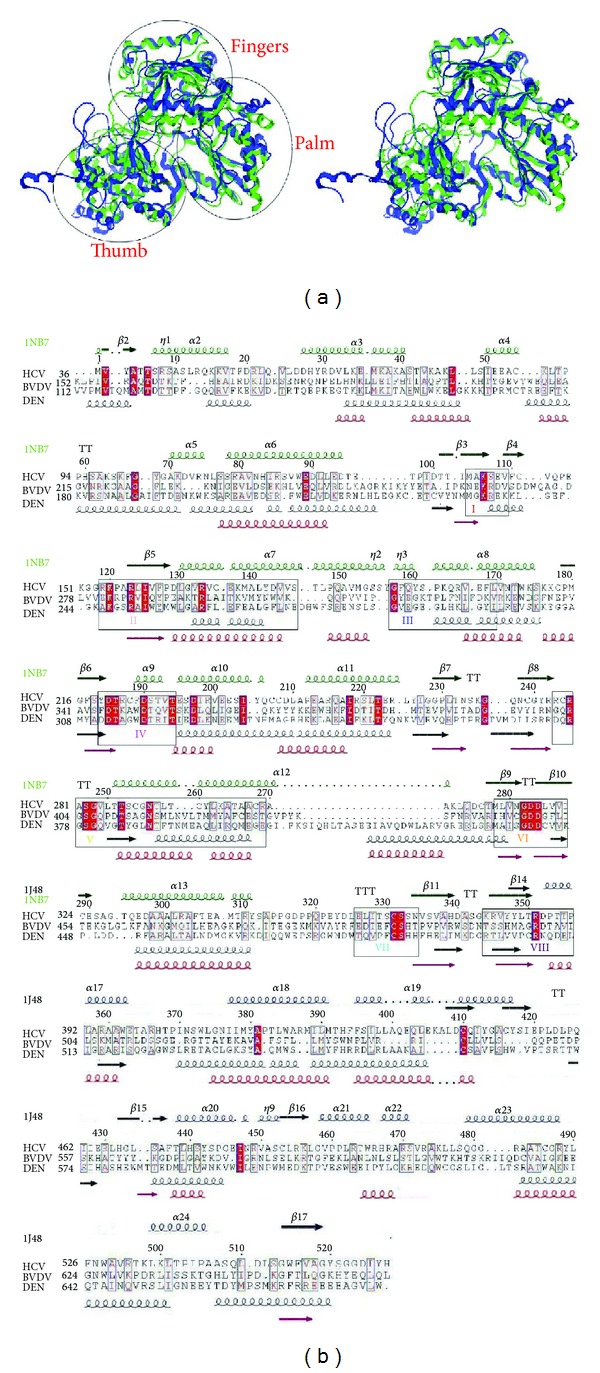
(a) Stereoview of the superimposed crystal structures of the HCV [7] (ribbons in green) and BVDV [8] (ribbons in blue) RNA-dependent RNA polymerases used as templates in the modelling procedure. (b) Sequence alignment of the HCV, BVDV, and Dengue polymerases derived as described in the text. Open and red shed boxes correspond to similarities and identities, respectively. Secondary structure elements corresponding to the template structures are given on the top of the alignment and colored in green for the HCV and in blue for the BVDV RdRp crystal structures. The secondary structure prediction for the Dengue polymerase and the actual secondary structure elements of the 3D model are shown below the alignment and are colored in black and red, respectively. The RdRp conserved motifs are boxed and labelled from I to VIII. The figure was produced using the ESPrint utility [9].

Towards the modelling of the 3D structure of the RNA-dependent RNA polymerase (RdRp) of Dengue virus (type II) the known crystal structures of the RdRps of hepatitis C virus (HCV) [27] and bovine viral diarrhoea virus (BVDV) [8], which also belong to the *Flaviviridae* family, were spatially aligned (Figure 1(a)). As described in Choi et al. [8] the regions comprising the “fingers” and “palm” domains share a high structural similarity between the two polymerases of this family as well as with RdRps from other families, whereas the region corresponding to the “thumb” domain is structurally distant following a different spatial arrangement relatively to “fingers” and “palm” domains. The sequence alignment resulting from a least-square minimization of structurally equivalent *C* ^*α*^ atoms between the two crystal structures of the *Flaviviridae* family is presented in Figure 1(b). 

The Dengue RdRp sequence was included to the above alignment guided by threading results through the program PHYRE [34] and the eight motifs, I to VIII, known to be conserved in all RdR polymerases [35]. As deduced by the sequence alignment (Figure 1(b)), the Dengue RdRp shared a relatively low overall sequence similarity with the two known RdRps: 34% sequence similarity (18% identity) with the HCV and a 31% sequence similarity (18% identity) with the BVDV RdRp, respectively. As expected, the sequence similarity originated mainly from the eight conserved motifs and key residues therein (Figure 1(b)).

### 3.2. Homology Modelling of the Dengue Virus RNA-Dependent RNA Polymerase

The modelling of the Dengue RdRp structure was based on the sequence alignment shown in (Figure 1(b)). The RCSB entries 1NB7 and 1S48 corresponding to the crystal structures of HCV and BVDV RdRps, respectively, were used as templates. The Dengue polymerase region comprising the conserved polymerase domains of palm and fingers was modelled based on the HCV structure, whereas the structurally variable “thumb” domain was modelled based on the BVDV structure. Although the later exhibited overall a higher sequence similarity with the Dengue polymerase, the first structure was preferred for the modelling of the structurally conserved polymerase region due to the presence of ssRNA in the HCV polymerase structure, which can be used to model the polymerase substrate—interacting sites with higher accuracy. 

**Figure 2:**
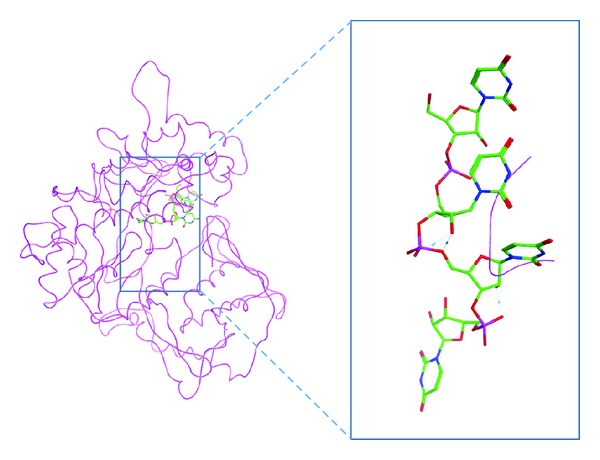
3D model of the Dengue RNA-dependent RNA polymerase produced by conventional homology modelling. This model exhibits only few secondary structure elements. Backbone clashes between the model and an ssRNA fragment (depicted in green sticks) copied from the HCV template structure indicate that the conventional homology modelling approach failed to model the ssRNA channel correctly. This figure demonstrates that conventional homology modelling of the Dengue polymerase was unsuccessful.

The model produced by the conventional homology modelling procedure showed only few secondary structure elements and was largely unstructured (Figure 2). In order to further evaluate the modelling procedure applied, the ability of the polymerase model to accommodate the substrate was investigated. The coordinates of the ssRNA from the HCV RdRp template were transferred to the model, for this purpose. The ssRNA fragment had numerous clashes with backbone atoms of a protein loop (Figure 2) indicating that the conventional method failed to predict the binding site correctly. This failure was mainly due to the low sequence similarity shared between the Dengue polymerase and the template structures used in the modelling procedure. 

### 3.3. 3D Space-Aided Homology Modelling

**Figure 3:**
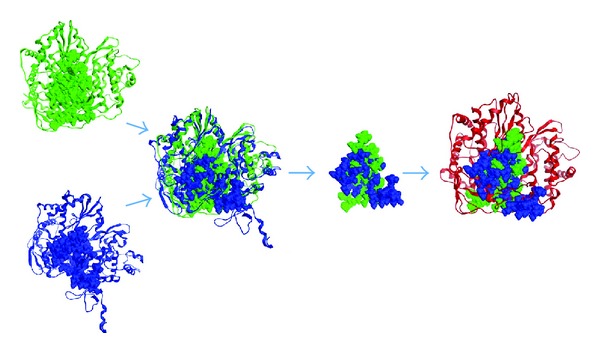
The novel (3D space-aided) approach to homology modelling. Left: the template structures of the HCV (in green) and BVDV (in blue) are shown in ribbon representation and their 3D space corresponding to the substrate channel is filled with alpha-spheres (see methods). Middle: the sum of alpha-spheres from the templates used to restrict the modelling of the Dengue polymerase. Right: the dengue polymerase model produced by the space-aided approach. The new model is enfolded up the sphere-filled cavity space of the templates, which guarantees that the space corresponding to substrates will not be occupied by parts of the protein.

In order to overcome the deficiency of the conventional homology modelling, a novel approach based on additional information from the template structures has been developed. The approach takes advantage of the space occupied by ligands or substrates in the template structures to restrain the folding of the target protein. In the case of the Dengue RdRp, the model was enfolded up the 3D conformational space corresponding to the channel occupied by the ssRNA, the Mn^++^ ions, and the rNTP tunnel in the template structures. For this purpose, the abovementioned 3D space was first filled with alpha-spheres (see Methods) in both templates (Figure 3). The sum of the sphere-filled cavities was subsequently used as a scaffold to restrain the folding of the model (Figure 3).

The quality of the produced model as assessed by PROCHECK [31] was similar to the quality expected for crystal structures determined at 2.9 Å. Namely, the Ramachandran plot quality assessment showed that 94.1–100% of the conformational *φ*,*ψ* angles of the model were located in allowed regions of the Ramachandran space, and the values of several geometrical parameters were comparable to typical values obtained from crystal structures determined at 2.9 Å (Table 1). 

### 3.4. Description of the 3D Space-Aided Model

**Figure 4:**
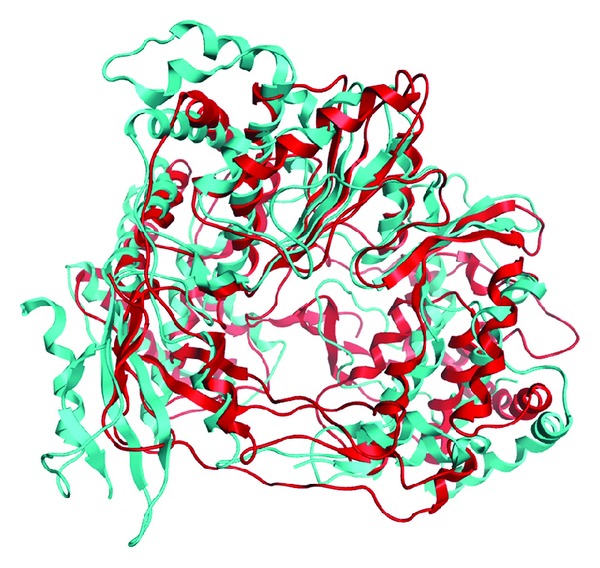
The Dengue polymerase model produced by the 3D space-aided approach (colour turquoise) superposed to the Dengue type II X-ray crystal structure (coloured red, PDB entry: 2BMF). All major *Flaviviridae *polymerase motifs have been structurally conserved in the Dengue polymerase model.

As expected from the sequence alignment (Figure 1(b)), the Dengue polymerase model produced by the novel approach exhibited the structural features of RdRps [36]. Namely, the three distinct domains of RdRps: “thumb,” “palm,” and “fingers” regions as well as the eight motifs (I–VIII) were structurally conserved in the Dengue polymerase model when structurally compared to the X-ray crystal protein structure of the same species (Figure 4). 

**Figure 5:**
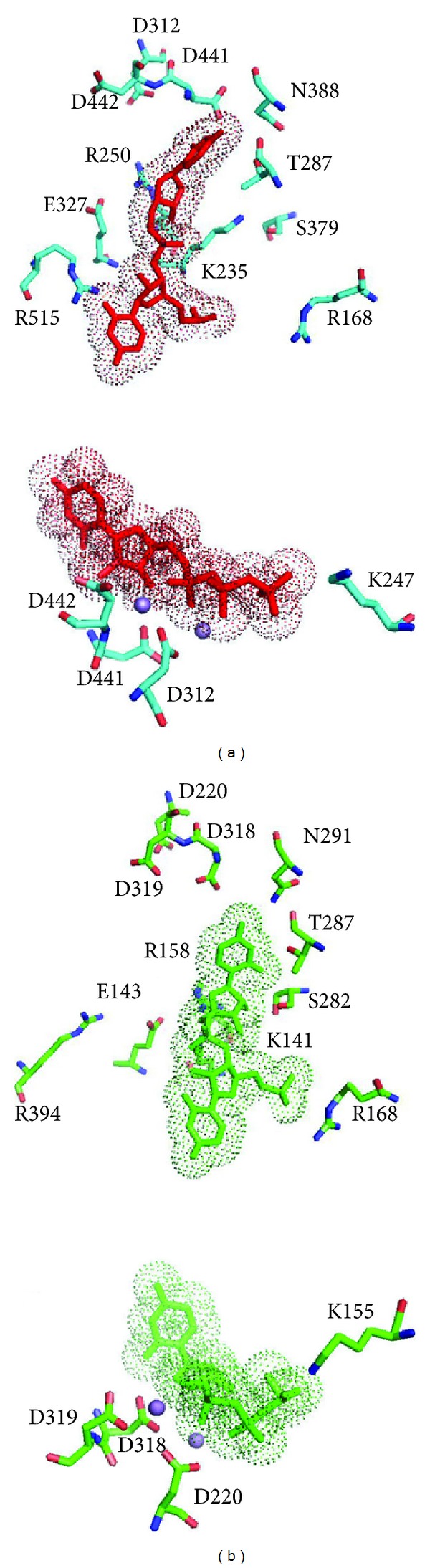
Closeup of the ssRNA (upper) and the UTP/Mn^++^ (lower) sites in (a) the dengue polymerase model produced by the 3D space-aided approach and (b) the Dengue X-ray polymerase structure, for comparison (PDB entry: 2BMF). Coordinates of the substrates were copied to the model from the template structure (see text). The substrates are depicted in sticks and dotted spheres, whereas spheres in magenta represent the catalytic Mn^++^ atoms. Invariant residues (labeled) of various motifs in the vicinity of either substrate are structurally conserved in this Dengue polymerase model demonstrating the efficiency of the novel approach as opposed to the conventional homology modeling.

In order to evaluate the substrate binding site, the model was subjected to energy minimization in the presence of the RdRp substrates. The coordinates of the ssRNA or UTP/Mn^++^ were transferred to the model from the HCV RdRp template structure (entries 1NB7 and1NB6, resp.) for this purpose. The model could accommodate either substrate upon energy minimization in contrast to the model obtained by conventional homology modelling. Invariant residues of various motifs in the vicinity of either substrate in the HCV template structure were conserved structurally in the Dengue polymerase model (Figure 5 and Table 3). Taken together these observations illustrated the viability of the novel approach to conventional homology modeling.

To evaluate further the novel approach, the models obtained by both conventional homology modelling and the 3D space-aided homology modelling method were compared with the X-ray resolved structure of the Dengue RdRp by calculating the root mean square deviations (RMSd) between equivalent atoms. Large RMSd values are indicative of systems of poor quality. The *C* ^*α*^ RMSd of the model produced by the novel approach from the equivalent domains of the template structures was less than 1.2 Å (Table 2). The low RMSd value indicated that this model remains conformationally close to the template structures even after minimization, reflecting its good quality. On the other hand, the *C* ^*α*^ RMSd of the conventionally built model from equivalent domains of the X-ray established Dengue RdRp structure was much higher (3.04 Å) (Table 2). These observations illustrated further the efficiency of the novel approach over conventional homology modelling.

### 3.5. Molecular Surface Analysis

**Figure 6:**
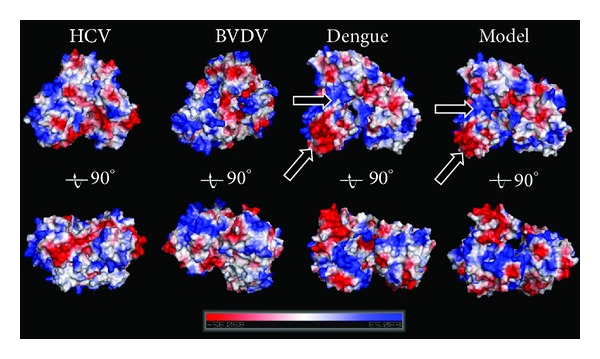
Surface comparison of the crystal structures of the HCV, BVDV, and Dengue RdRp (PDB entry: 2BMF) versus the 3D model of the Dengue polymerase that was produced by the 3D space-aided homology modelling approach. The surfaces are colored according to their electrostatic potential.

In order to analyze the molecular surface of the produced Dengue polymerase model, the electrostatic potential surface of the 3D space-aided model was calculated (see Section 2). For direct comparison the electrostatic potential surfaces were also calculated for the X-ray-established structures of HCV, BVDV, and Dengue RdRp that were used in this study. The X-ray structure of Dengue and the space-aided model shared common features such as a negatively charged rNTP tunnel and a widely positively charged surface in the vicinity of the rNTP channel entrance (Figure 6). 

### 3.6. Comparison of the Modelled RdRp with the X-Ray Crystal Structure

The 3D crystal structure of Dengue has been determined by X-ray crystallography. Therefore, a direct comparison can be performed between the homology model and the crystal structure of the Dengue RdRp. The ultimate aim is to judge whether the space-aided homology modelling approach did make a significant difference and improvement to the conventionally built model using unrestrained and unbiased homology modelling. The RMSd between the two models is 4.6 Å. The RMSd between the conventionally built and the space-aided RdRp models is 6.3 Å and 1.4 Å respectively. The 3D structure of the space-aided model is very similar to that of the crystal structure (Figures 4 and 6). On the other hand it was found that conventional homology model produced a not-so viable 3D model. There were many bumps and regions of low similarity to the crystal structure (Figure 2). Most importantly, the Ramachandran plot of the space-aided model was the better one, since there were no residues in the disallowed areas of the plot (data not shown). A structural superposition of the crystal structure and the two models is summarized in Figure 4. 

## 4. Conclusions

In the current study, a novel approach to conventional homology modelling has been developed. The approach takes advantage of the 3D conformational space corresponding to the template's shape and size characteristics as well as the existence of ligands and substrates, which are used to restrain the folding of the target (query) protein. With the example of the successful modelling of the 3D structure of the RNA-dependent RNA polymerase of Dengue (type II) virus, which shared low sequence identity with the chosen templates, the new approach illustrated an efficient way to model 3D structures of enzymes sharing low sequence identity with the modelling templates.

## Figures and Tables

**Table 1:** Parameters reflecting the quality of the Dengue polymerase model calculated by PROCHECK [25]. Parameter values represent observed values for the Dengue polymerase model produced by the cavity space-aided approach compared to typical values obtained refined structures at 2.9 Å.

Stereochemical parameters	Number of data points	Parameter value	Typical value	Band width	Number of band widths from mean	Quality compared to structures at 2.9 Å
A: main chain parameters						
Percentage residues in A, B, L	490	71.0	68.7	10.0	0.	Inside
Omega angle st dev	545	9.0	6.0	3.0	1.0	Inside
Bad contacts/100 residues	0	0.0	17.9	10.0	−1.8	BETTER
Zeta angle st dev	508	3.3	3.1	1.6	0.1	Inside
H-bond energy st dev	290	0.7	1.0	0.2	−1.7	BETTER
Overall G-factor	547	−1.0	−0.8	0.3	−0.9	Inside

B: side chain parameters						
Chi-1 gauche minus st dev	86	11.5	26.3	6.5	−2.3	BETTER
Chi-1 trans st dev	168	11.3	25.7	5.3	−2.7	BETTER
Chi-1 gauche plus st dev	203	12.1	24.3	4.9	−2.5	BETTER
Chi-1 pooled st dev	457	12.9	25.1	4.8	−2.5	BETTER
Chi-2 trans st dev	109	13.5	25.3	5.0	−2.3	BETTER

**Table 2:** Summary of sequence and structural similarities between the homology models of dengue polymerase (novel and conventional approaches) and the RdRp known structures (RCSB codes in parenthesis) used as templates.

Template	Sequence comparison			RMSd (Å)		
	Number of equivalent amino acids	Identity (%)	Similarity (%)	Number of atoms	Novel	Conventional
HCV RdRp (1NB7)	351	18	34	139 (*C* ^*α*^)	1.19	3.57
BVDV RdRp (1S48)	172	18	31	65 (*C* ^*α*^)	1.04	3.04

**Table 3:** Equivalent invariant residues between the HCV template and the Dengue virus polymerase model in the vicinity of the ssRNA fragment or the UTP substrate.

Substrate	ssRNA			UTP		
Interacting invariant RdRp residues	HCV RdRp structure	Dengue virus Model	Motif	HCV RdRp structure	Dengue virus Model	Motif
	D220	D312	IV	D318	D441	VI
	D318	D441	VI	D319	D442	VI
	D319	D442	VI	D220	D312	IV
	N291	N388	V	K155	K247	II
	T287	T384	V			
	R158	R250	II			
	E143	E327	I			
	S282	S379	V			
	K141	K235	I			
	R168	R260	II			
	R394	R515	Conserved			
